# Household food insecurity and associated factors among postpartum women in southern Ethiopia: a community based cross sectional study

**DOI:** 10.1038/s41598-024-54666-w

**Published:** 2024-02-18

**Authors:** Dagne Deresa Dinagde, Habtamu Wana Wada, Menen Tilahun Chewaka

**Affiliations:** 1 Departments of Midwifery, College of Health Sciences, Mattu University, Mattu, Ethiopia; 2 Departments of Midwifery, College of Medicine and Health Sciences, Arba Minch University, Arba Minch, Ethiopia; 3 Departments of Midwifery, College of Medicine and Health Sciences, Mizan Teppi University, Mizan, Ethiopia

**Keywords:** Antenatal care, Arba Minch town, Food insecurity, Postpartum, Southern Ethiopia, Health care, Nutrition

## Abstract

Approximately two billion individuals, or 26.4% of the global population, live in moderate- to severely food-insecure circumstances. It was discovered that not receiving all recommended antenatal care throughout one's pregnancy is one of the effects of household insecurity. The majority of women in Ethiopia, one of the most food-insecure countries in the world, with 10% of the population facing food poverty, devote more of their time to household duties, making food insecurity in the home the primary effect of poor prenatal care utilization. The main objective this study was to assess the status of household food insecurity among postpartum women at Arba Minch town, southern Ethiopia**.** A community-based cross-sectional study was conducted among 381 mothers who were enrolled from December 1, 2022, to January 30, 2023. The total sample size was allocated proportionately to the number of postpartum women living in each kebeles taking from the town registry of health extension workers for immunization. Thus, systematic sampling was applied. Kobo Toolbox was used for data collection and cleaning, which was then analyzed using the statistical package of Social Science Version 26 (SPSS). In this study, the prevalence of food insecurity was 30.2% (95% CI 25.5–34.5). The associated factors with household food-insecurity were maternal occupation (AOR = 0.5, 95% CI 0.27, 0.90), late antenatal care initiation (AOR = 3.5, 95% CI 2.13, 5.91), and low monthly income (AOR = 3.1, 95% CI 1.38, 6.93). Food insecurity among postpartum mothers in the study area is high. Families who are severely food insecure require quick assistance to lower poor maternal and neonate’s outcomes. Furthermore, enhancing the occupation of mothers is crucial in reducing the morbidities and mortality of food insecure mothers, such as delayed prenatal care services, anemia, low birth weight, and stillbirth.

## Introduction

Food insecurity is still a significant global development and public health issue that negatively impacts people's productivity, well-being, and frequently even their ability to survive^1,2^. Poor health and a drop in productivity are two additional issues linked to food insecurity that households with limited access to food frequently confront. This can frequently result in a vicious pattern where households fail to produce enough food^3^.

Globally, about two billion individuals, or 26.4% of the world population, live in moderate- to severely food-insecure circumstances with the majority, 676 million (34%) are living in Africa, 188 million (9%) in Latin America and 1.04 billion (52%) are in Asia. These individuals are at a higher risk of malnutrition and ill health due to their infrequent access to sufficient and nourishing food^2,4^. Africa has the highest rate of overall food insecurity in the world, impacting over half of the population, with varying effects throughout the continent's regions: the southern region accounts for 53.6%, the eastern region for 62.7%, and the western region for 47.9%^5,6^. 10% of the population of Ethiopia experiences chronic food-insecurity, making it one of the most food insecure countries in the world^7^. According to research from a rural district in the country's north, in 70.7% of households were food insecure and this situation was comparable with 75% of households experiencing food insecurity in Addis Ababa^8^.

One consequence of coming from a household with inadequate food was found to be not receiving all of the recommended prenatal care during one's pregnancy^9,10^. Pregnant women receive specialized care known as antenatal care (ANC) through public services with the goals of preventing diseases from the mother and the unborn baby, promoting the wellbeing of the mother and the fetus through early detection and prompt treatment of pregnancy-related complications^11,12^. Thus, it was able to decrease the maternal mortality ratio by about 44% in the last 25 years, from 385 (359–427) in 1990 to an estimated 216 (80% uncertainty band 207–249 maternal deaths per 100,000 live deliveries in 2015^13^.

A few studies have been done on how the use of antenatal care services was affected by household food security status in Ethiopia. To the best of the researchers' knowledge, however, the information that was available indicated that antenatal care attendance was related to factors such as income/wealth status, educational level, unplanned pregnancy, awareness of antenatal care, husband/maternal occupation, marital status, housing, and food insecurity^9,10,14,15^.

Prior studies have also demonstrated the impact of neighboring socioeconomic pressures, such as poverty and food insecurity, on how people use health care^16^. Women in developing countries, such as Ethiopia, put in a lot more time and effort caring for the home than seeking medical care. As a result, when women struggle to obtain food, they must choose between meeting their nutritional needs and access to healthcare, which may lead to inappropriate or underuse of healthcare services in general and maternal healthcare in particular^9,17^.

Given the severity of the issues and the dearth of relevant data in Ethiopia, this study hypothesized that household food insecurity might be one of the factors preventing people from attending antenatal care and observed its independent association. There was little evidence linking the use of prenatal care to pregnant women's access to food security. In filling this gap in the scientific literature, the discovery is predicted to have a significant impact on the nation's public health policy-making. Hence, the aim of this study was to assess the food insecurity status among postpartum women in Arba Minch town, southern Ethiopia.

## Methods and materials

### Study design and setting

**Figure 1:**
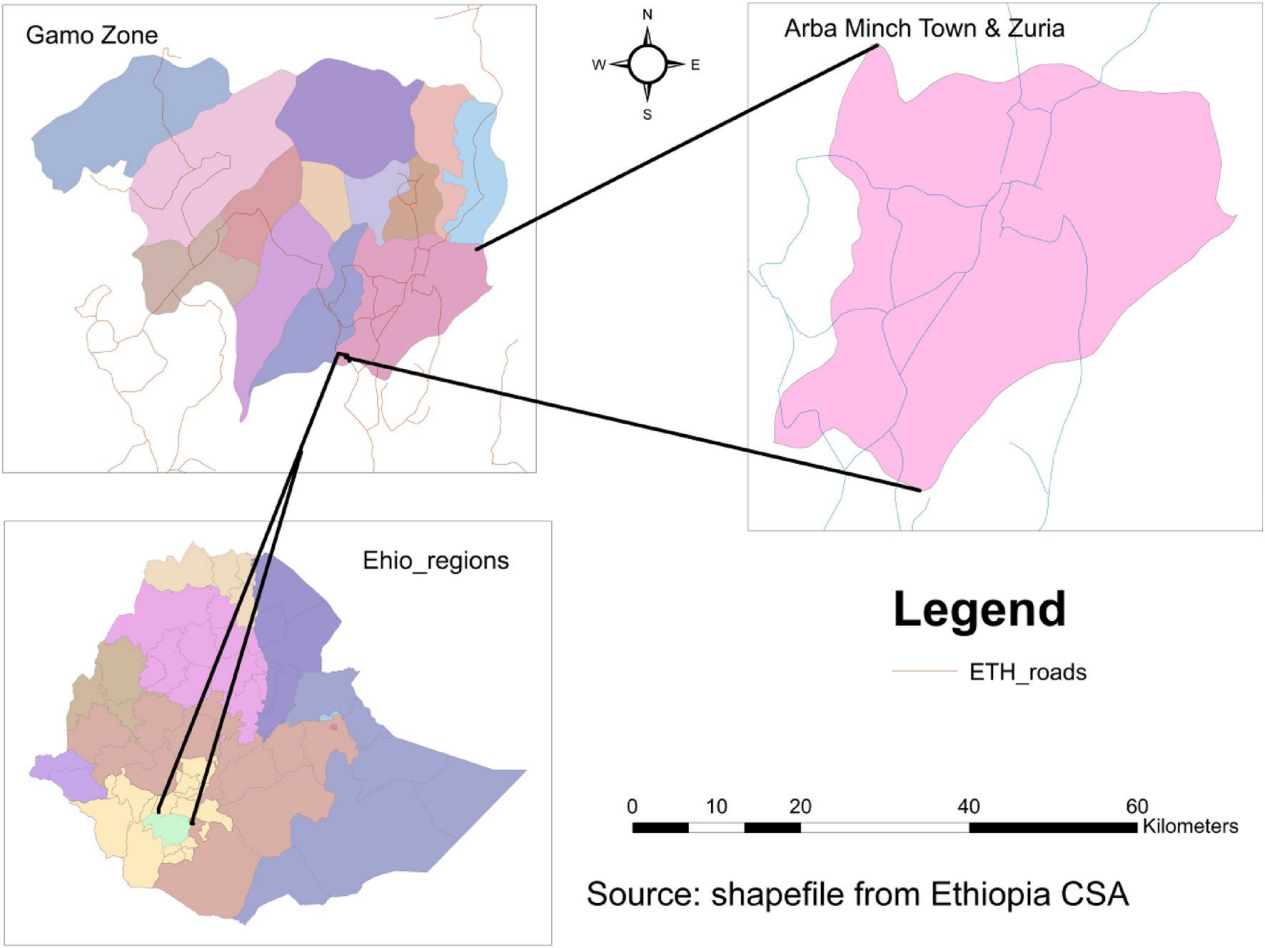
Map of the study area [Shape file source: CSA, 2013; URL: https: //www.africaopendata.org/dataset/ethiopia-shapefiles].

A community-based cross-sectional study was conducted among postpartum women who gave birth within six months ago, from December 1, 2022, to January 30, 2023, in Arba Minch town (Fig. 1). The town is situated 275 km from Hawassa, the commercial and administrative hub of the southern area, and 505 km southwest of Addis Ababa, Ethiopia's capital city. According to the 2022 population projection, Arba Minch town has a total population of 201,049 with 100,019 females and 101,130 males^18^. Two governmental hospitals, one private hospital, and two health centers serve the community. Antenatal care is offered in all medical facilities. In those healthcare institutions, there are 30 midwives and 15 nurses who provide pregnancy care. More than 500,000 people in the town and surrounding areas are anticipated to receive public health services from the local facilities^19^.

### Study participants

The study participants were those postpartum women who gave birth within six months ago in Arba Minch town and were available during the data collection period. Those participants willing to give information were selected by systematic random sampling, and those mothers who were critically ill on the day of data collection and those who were not constantly living in Arba Minch town were excluded from the study.

## Sample size determination and sampling technique

The sample size was calculated using single population proportion formula:$n={(za)}^{2}\frac{p(1-p)}{{{\varvec{d}}}^{2}}$, considering the following assumptions: prevalence of food insecurity among pregnant women was taken from study conducted in Jimma zone, southwest Ethiopia (9%)^20^ with confidence interval of 95% and, 3% margin of error.

$$n={(za)}^{2}\frac{p(1-p)}{{{\varvec{d}}}^{2}}$$

$$n={(1.96)}^{2}\frac{0.09(1-0.9)}{{0.03}^{2}}=346$$

Finally, 381 mothers were included in this study by adding 10% non-response rate.

**Figure 2:**
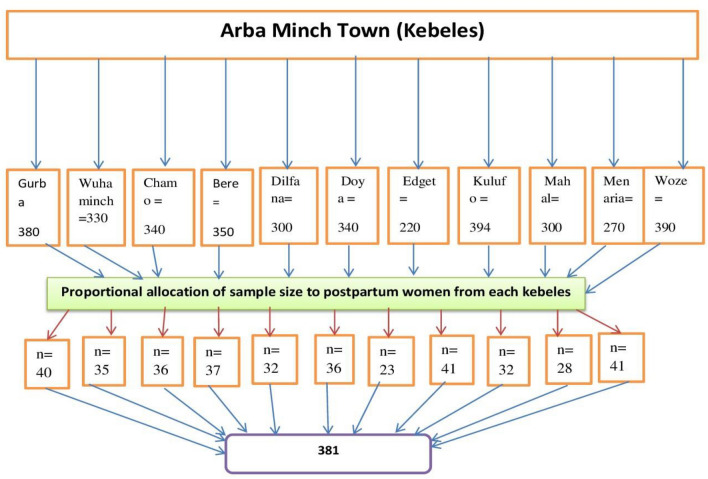
The schematic diagram shows proportional allocation of sample size of each kebeles of Arba Minch town.

There were four sub-cities in the town which were divided into 11 Kebeles (small administrative unit of Ethiopia). The sample size was allocated to all Kebeles proportionally to their postpartum size. Each mother was selected by a systematic random sampling technique using the town registry of the local health extension workers for immunization as a sampling frame. See (Fig. 2). Then the proportion of eligible mothers those who gave birth in the last six months prior to this study was identified. Then individual participants who were going to be interviewed first were known by the lottery method from one to the sample size of the appropriate kebele. The subsequent household to be included in the study was interviewed at the kth interval through a house-to-house visit. For households with more than one eligible woman, an interview was done for one of the mothers using a simple random sampling technique. If an eligible woman lived in that house at the time of the survey but was not there, it was revisited two to three times.

### Study variables

#### Dependent variable

Household food insecurity (Yes/No).

#### Independent variables

**-Socio-demographic factors: **Age, residence, maternal education, maternal occupation, husband education, Income, number of children, head of households, media exposure.

**-Obstetrics related factors: **Time of antenatal visit, number of antenatal care contact, and knowledge of antenatal care services.

### Operational definition

**Food insecurity**: According to the World Food Summit of 1996, food security is the state in which all people, have physical and financial access to enough safe, nourishing food that satisfies their dietary needs and food choices for an active and healthy life and vice versa in food insecurity^21^. The Household Food Insecurity Access Scale (HFIAS), developed by the Food and Nutrition Technical Assistance (FANTA) Program of the United States Agency for International Development, will be used to assess household food security. It consists of nine items and in this study, household food security status was divided into two categories: "food secure" when participants did not experience any food access conditions in the previous four weeks, and "food insecure" when they were unable to access sufficient food at all times to lead an active and healthy life^22^.

**Poor utilization of ANC**: Women are regarded as having dropped out of prenatal care if they did not attend each suggested visit according to new WHO recommendation or on the other hand, it included those who delayed registration of ANC or discontinued the services^23^.

## Data quality management

Following an extensive review of relevant literature and similar studies^7,17,20,24,25^, a data collection tool was developed to ensure the quality of the data. Properly designed data collection instruments were provided after two days of training for data collectors and supervisors. Pre-testing of the questionnaire was carried out two months before the commencement of the data collection among 20 postpartum mothers living in Mirab Abaya, the nearest study town, and all the necessary corrections were made based on the pretest result to avoid any confusion and for better completion of the questions. Every day, the collected data were reviewed and cross-checked for completeness and relevance. Checking for double data entry, consistency, missing values, and outliers was done by the supervisors and principal investigator, and comments and measures were undertaken throughout the data collection period.

### Statistical analysis and entry

The data was coded, collected, cleaned, and entered by Kobo Toolbox and exported to the statistical package for social science (SPSS) version 26 for analysis. Inconsistencies and missing values were checked by running frequencies and other data explorations. Descriptive statistics like frequency distributions, mean, and standard deviation were computed. Bivariate analysis was done primarily to check which independent variables had an association with the dependent variable. Independent variables with marginal associations (*P* < 0.25) in the bivariate analysis, which are biologically plausible and showed significant association in the previous studies, were entered into a multivariate logistic regression analysis in order to detect association with household food insecurity (HFIAS). The multicollinearity was checked among independent variables, and the Hosmer–Lemeshow test was used to check the appropriateness of the model for analysis. Finally, adjusted odds ratios (AOR) with 95% CI were estimated to assess the strength of associations and statistical significance was declared at a *P* value < 0.05^26,27^ and results will be presented using tables, figures, and texts.

## Results

### Socio-demographic characteristics of study participants

All participants (381) were actually interviewed and provided accurate information, yielding a response rate of 100%. The mean age of the women was 29.7 years (SD ± 6) with a minimum and maximum age of 18 and 42, respectively. The majority of the participants, two hundred one (52.8%), were Orthodox Christians. Most women, three hundred (78.3%), can read and write. The majority of the women, one hundred eighty-one (47.5%), were housewives and about three hundred nine (81.1%) of their husbands can write and read. Three-fourth, of participants reported that their household head was their husband. The majority, three hundred twenty-two (84.5%), of the study participants have a monthly income of more than five thousand Ethiopian birr (Table 1).

**Table 1:** Socio-demographic characteristics of study participants at Arba Minch town, 2023 (n = 381).

Variables	Response	Frequency	Percent (%)
Age (in years)	< 20	6	1.6
	20–24	82	21.5
	25–29	6	1.6
	30–34	82	21.5
	35 and above	119	31.2
Residence	Rural	121	31.8
	Urban	260	68.2
Maternal education	Unable to read and write	81	21.3
	Able to read and write	154	40.4
	Primary	87	22.8
	Secondary school & above	59	15.5
Maternal occupation	civil servant	75	19.7
	Farming	22	5.8
	house wife	181	47.5
	Traders	103	27.0
Husband education	Unable to read and write	72	18.9
	Able to read and write	163	42.8
	Primary	60	15.7
	Secondary school & above	86	22.6
Income	< 2500 ETB	11	2.9
	2501–5000 ETB	48	12.6
	> 5000	322	84.5
Number of children	≤ 3	19	5
	4–6	343	90
	≥ 7	19	5
Head of households	Husband	255	66.9
	Herself	126	33.1
Media exposure	Yes	318	83.5
	No	63	16.5

### Obstetrics related factors

Among a total of 381 participants who had given birth in Arba Minch town and lived in the town, almost all of them (94%) could show their ANC appointment card, and nearly half (47.5%) started antenatal care early within three months of pregnancy. Out of those who started ANC early, within three months of pregnancy, about 75% of participants received all recommended ANC visits according to the new WHO recommendation.

### Food security status of households

**Figure 3:**
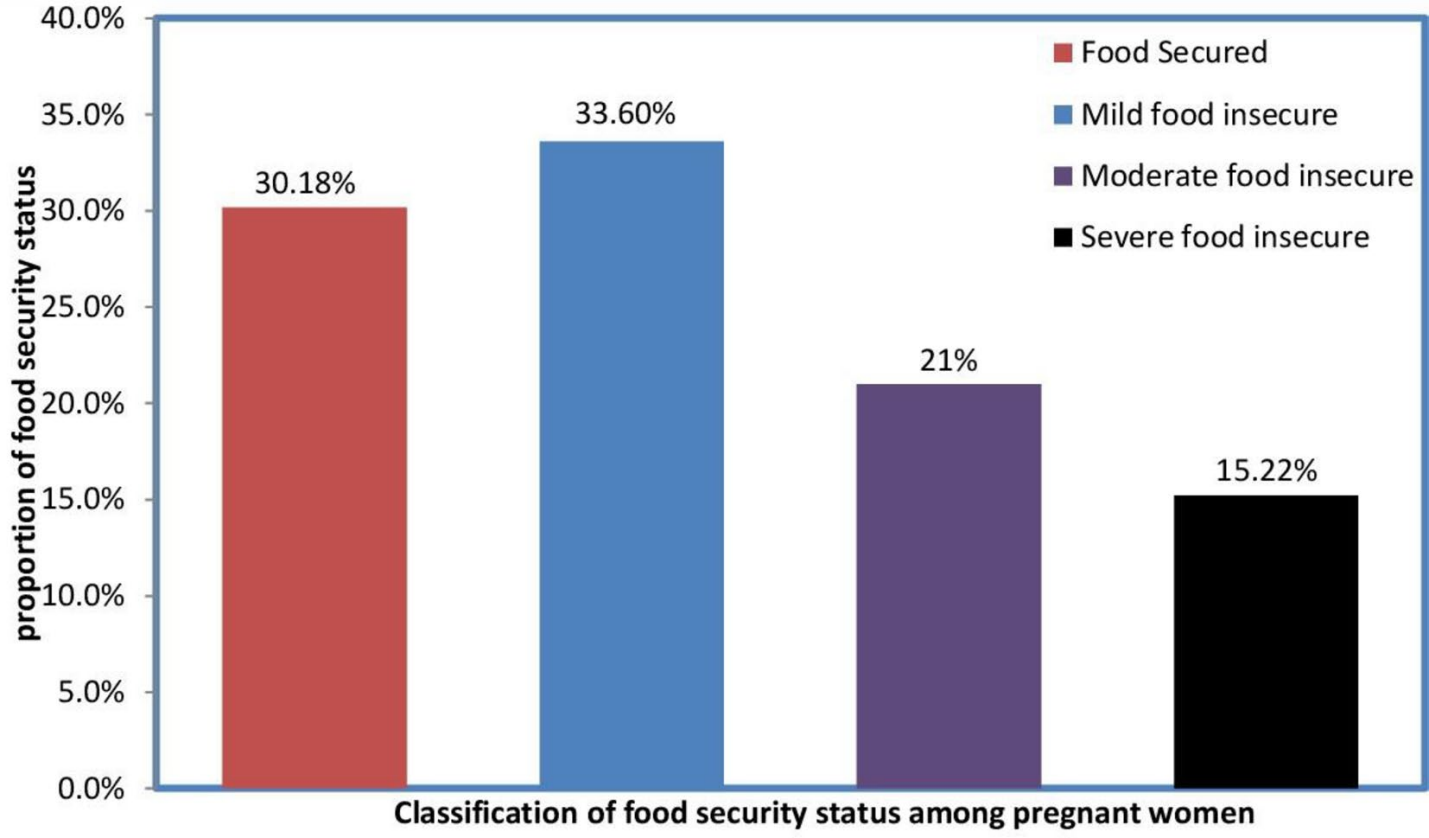
Household’s food security status of postpartum mothers of Arba Minch town, southern Ethiopia, 2023 (n = 381).

Findings of this study indicated that out of 381 participants approximately 115 (30.2%) with 95% CI: 25.5–34.5 of households were food insecure **(**Fig. 3**)** with approximately 128 (33.6%) 80 (21%) and 17(15.2%) were experienced mild, moderate and severe food insecurity respectively. of the respondents were food secure while and of respondents were mildly and severely food insecure respectively. Of the total households, 100 (29.9%) of them reported that they have ever experienced sleeping hungry and 98 (30.2%) had no any kind of food in the house at the time of the survey. 154 (40.4%) households had just a few kinds of foods while 190 (50%) households reduced the amount of meal that they consumed.

### Factors associated with household food insecurity

In the bivariate binary logistic regression, those variables with *P* < 0.25 were candidates for multiple logistic regressions, and statistical significance was declared at a *P* value < 0.05 as below. Thus, age, maternal education, maternal occupations, husband education, husband occupation, income, head of household, exposure to media, occupation of household head, family size and timely initiation of ANC, were candidates for multivariate.

The odds of food insecurity among women earning low income were three times more likely as compared with those earning more monthly income (> 5000 ETB) (AOR = 3.1, 95% CI 1.38, 6.93).

Those mothers working on trading were 50% less likely to face food insecurity when compared to those mothers employed to government or non-governmental agents (AOR = 0.5, 95% CI 0.27, 0.90). The odds of food insecurity among women were about 3 times more likely among those mothers to receive ANC visits later as compared with those who received ANC early (AOR = 3.5, 95% CI 2.13, 5.91) (Table 2).

**Table 2:** Multivariate logistic regression analysis result for variables associated with household food insecurity at Arba Minch town, 2023 (N = 381).

Variables	Responses	Household food insecurity		COR (95% CI)	AOR (95% CI)	*P* value
		Yes (%)	No (%)			
Maternal occupation	Farming	38 (50.7)	37 (49.3)	1.76 (0.96, 3.22)	1.77 (0.93, 3.36)	0.081
	House wife	7 (31.8)	15 (68.2)	0.8 (0.3.0, 2.13)	0.71 (0.25, 2.07)	0.58
	Traders	32 (17.7)	148 (82.3)	0.35 (0.21, 0.64)	0.5 (0.27, 0.90)	0.021*
	Civil servant	38 (36.9)	65 (65.1)	1	1	–
Monthly income	< 2500ETB	19 (55.9)	15 (44.1)	3.1 (1.48, 6.25)	3.1 (1.38, 6.93)	0.06*
	2500-5000ETB	8 (16.7)	40 (83.3)	0.5 (0.22, 1.10)	0.64 (0.27, 1.52)	0.321
	> 5000ETB	88 (29.4)	211 (69.6)	1	1	0.010*
Family size	≤ 3	7 (36.8)	12 (63.2)	1	1	0.321
	4–6	106 (30.9)	237 (69.1)	0.77 (0.29, 2.0)	4.01 (0.61, 27.3)	0.14
	≥ 7	5 (12.5)	14 (87.5)	0.2 (0.40, 1.14)	2.4 (0.48, 12.17)	0.280
Time of ANC visit	Late/miss	86 (43)	114 (67)	3.95 (2.43, 6.43)	3.5 (2.13, 5.91)	0.000*
	Early	29 (16)	152 (84)	1	1	–

* = p < 0.05 (statistically significant), 1 = Reference group (unexposed group).

## Discussion

This study found that the prevalence of household food insecurity among postpartum women in study area was 30.2%. This level of food insecurity is lower than the level of food insecurity among general population which is 35%^28^. This finding was in line with study conducted at Debra Berhan town (32.4)^29^. However, this funding is much lower than studies conducted in Jimma town among pensioners (83.5%)^24^, Sidama district (82.3%)^30^ and Ari district southern Ethiopia (44.8%)^31^. Because all of the respondents were expecting, they may have received extra preparation for their pregnancy time, and households may have readily accessed assistance from their partners through specific care throughout pregnancy, which may have contributed to the decreased degree of food insecurity shown in our study. According to this study, household food insecurity was reported by one-third, one-fourth, and almost one-seventh of study participants, respectively, ranging from mild, moderate and severe.

In keeping with Sustainable Development Goal 2 (which strives to end hunger, achieve food security, and improve nutrition) and Sustainable Development Goal 8, which promotes economic growth and productive employment for all, maternal employment benefits women both socially and economically^32^. Thus, those mothers working on trading were 50% less likely to face food insecurity when compared to those mothers employed to government or non-governmental agents (AOR = 0.5, 95% CI ( 0.27, 0.90). This may be acceptable given that merchants are able to support their families and themselves with more daily money. Additionally, this could be because of the daily activities involved in trading, which prevents parents from being at home to feed their family with enough food. This finding is supported by study conducted at Jimma town, Ethiopia^24^.

According to this study, about 84% of women from food-secure households reported that they had visited health care facilities for antenatal care before 12 weeks. It was found that food insecurity was a separate determinant of how often mothers sought medical attention. The odds of food insecurity among women were about three times more likely among those mothers to receive antenatal care visits later as compared with those who received ANC early (AOR = 3.5, 95% CI 2.13, 5.91). The fact that women in impoverished nations like Ethiopia spend more time on home responsibilities than on their health may help to explain this. Because food demand outweighs healthcare demand, women in homes experiencing food insecurity may decide against obtaining ANC. This Finding is supported by studies conducted among the poorest Americans^17^ and southern Ethiopia^9^.

About 56% of women with an average monthly income of less than 2500 ETB experienced food insecurity. Only 17% and 29% of middle-income households (2500–5000 ETB) and greater than 5000 ETB exposed to food insecurity, respectively, during this survey. Women with low income (< 2500 ETB) had a three times greater likelihood of experiencing food insecurity than those with higher monthly incomes (> 5000 ETB) (AOR = 3.1, 95% CI 1.38, 6.93). Reduced employment options, decreased incomes, and supply chain disruptions for low-income individuals (such as those who work as day laborers, rickshaw pullers, hotel employees, and street vendors) pose a threat to the improvement of food insecurity. Furthermore, the reason behind this is that women with higher incomes can combat food insecurity by having a backup source of food. This finding was consistent with studies conducted at north Shewa^33^ and Farta district, northwest Ethiopia^8^.

### Strength and limitation of the study

Given that the HFIAS measures food insecurity based on a self-reported answer, it may be subject to social desirability bias. However, prejudice could exist in both directions. If the respondents lack confidence in discussing their food security issues with others, it may also understate the severity of food insecurity. Furthermore, there is very little chance of bias because the scale has been confirmed in Ethiopia and other impoverished nations. The current predictors of food insecurity may not necessarily have a cause-and-effect relationship with food insecurity because the study was cross-sectional in nature.

## Conclusion and recommendation

This study showed that the food insecurity among mothers in this area was high. Late initiation of ANC, maternal occupation, and monthly income were independently associated with food insecurity. Families who are severely food insecure require quick assistance to lower mortality and morbidity. Additional initiatives and programs should think about ways to raise households' monthly incomes, such as by creating better jobs for people.

### Recommendation

**For Zonal job creation and vacation department:** Enhancing the occupation of mothers is crucial in reducing the morbidities and mortality of food insecure mothers, such as delayed prenatal care services, anemia, low birth weight, and stillbirth. Priority should be given to those with low monthly incomes or those who are unemployed full-time.

**For zonal and woreda health office:** Health care professionals should put in a lot of effort to encourage and give family planning, which is indirectly extremely beneficial in having planned family members based on their monthly income and capacity to feed their kids.

**For policy makers:** In order to improve livelihoods, additional interventions and programs should take into account ways to increase household members' participation in a variety of income-generating activities and boost agricultural productivity by utilizing services provided by productive safety net programs and agricultural extension.

**For feature researchers:** It is our recommendation that researchers focus on identifying the types of diets that are mostly lacking in Ethiopia at molecular level (vitamins and minerals) and the long-term effects of food insecurity.

### Ethical approval and consent to participate

The Arba Minch University College of Medicine and Health Science's institutional review board granted ethical clearance with reference number IRB/DD 1328/2022 and the formal letter was sent to all of the town's local health post. Prior to enrollment, participants were made aware of the study's purpose and importance. Before any data was collected, each participant gave their verbal informed consent. This approach was approved by the institutional review board due to the fact that some study participants are not literate. All study-related materials did not contain any personal information, and privacy was ensured. Everybody was free to participate. Study was conducted according to regulations and guidelines for researches involving human beings.

### Supplementary Information

Supplementary Information.

## Data Availability

The data used and/or analyzed are with correspondence author and is available upon reasonable request.

## Abbreviations

ANC: Antenatal care
HFIAS: Households Food Insecurity Assess Scale
SDG: Sustainable Development Goals
