# Supplementary material for: Household food insecurity and associated factors among postpartum women in southern Ethiopia: a community based cross sectional study
Source: Sci Rep. 2024 Feb 18;14:4003. doi: 10.1038/s41598-024-54666-w (PMC10874939; doi:10.1038/s41598-024-54666-w)
Supplement: Supplementary file 1 — Supplementary Information. [file 41598_2024_54666_MOESM1_ESM.docx]

| NO | **Socio-demographic factors** |  |
| --- | --- | --- |
| 101 | How old are you? | In years ……………….. |
| 102 | Residence | 1. Rural 2. Urban |
| 103 | Household Monthly income | ---------------ETB |
| 104 | Your religion? | 1. Orthodox 2. Muslim 3. Protestant   Others specify……….. |
| 106 | Your educational level? | 1. Unable to read and write 2. Able to read and write 3. Primary 4. Secondary school & above |
| 107 | Your occupation? | 1. House wife 2. Farming 3. Traders 4. Civil servant   Other specify …………. |
| 108 | Your marital status? | 1. Single 2. Married but Separate 3. Married and live to together 4. Divorced 5. Windowed |
| 109 | If married, Your husband’s educational status? | 1. Unable to read and write 2. Able to read and write 3. Primary 4. Secondary school & above |
| 110 | If married, Your husband’s occupation? | 1. Farming 2. Traders 3. Civil servant 4. Other specify … |
| 111 | Family size | ----------- |
| 112 | Sex of household head | 1. Father 2. Mother 3. Other --------- |
| 114 | Occupation of husband/cohabitant male partner | 1. House wife 2. Farming 3. Traders 4. Civil servant   Other specify …………. |
|  | **Obstetric related factors** |  |
| 1 | Time of ANC visit for first time during current pregnancy? | In months------------ |
| 2 | Have you received all recommended ANC visits? | 1. Yes 2. No |
| 3 | Please, would you show your ANC appointment card? | 1. Yes 2. No |
| 4 | If yes to question no 3 answered ‘yes’ record number of visits | -------------- |
| 5 | Parity |  |
|  | **Food Insecurity (Ask in the past four weeks)** |  |
| 001 | Are you worried about food shortage during last 4 wk.? | 1.No  2. yes [1. rare  2. sometimes  3. often] |
| 002 | Can all your family eat the quantity of food they want? | 1.No  2. yes [1. rare  2. sometimes  3. often] |
| 003 | Were you or any household member not able to eat the kinds of foods you/they preferred because of a lack of resources? | 1.No  2. yes [1. rare  2. sometimes  3. often] |
| 004 | Did you or any household member have to eat a limited variety of foods due to a lack of resources | 1.No  2. yes [1. rare  2. sometimes  3. often] |
| 005 | Did you or any household member have to eat some foods that you really did not want to eat because of a lack of resources to obtain other types of food? | 1.No  2. yes [1. rare  2. sometimes  3. often] |
| 006 | Did you or any household member have to eat a smaller meal than you felt you needed because there was not enough food? | 1.No  2. yes [1. rare  2. sometimes  3. often] |
| 007 | Was there ever no food to eat of any kind in your household because of lack of resources to get food? | 1.No  2. yes [1. rare  2. sometimes  3. often] |
| 008 | Did you or any household member go to sleep at night hungry because there was not enough food? | 1.No  2. yes [1. rare  2. sometimes  3. often] |
| 009 | Did you or any household member go a whole day and night without eating anything because there was not enough food | 1.No  2. yes [1. rare  2. sometimes  3. often] |
